# Keep Thinking

**Published:** 1899-02

**Authors:** Austin Bierbower


ARTICLE VIII.

### KEEP THINKING.

Keep thinking. What is not done with thought comes by chance;—and there are too many possibilities for the best to follow accident. When things may be a hundred ways there is small chance that they will fortuitously be the best. We hope only from design; and there should be as much thought as action.

When one goes not by thought he goes by nothing; and when chance takes him he arrives nowhere. He should not give himself over to nothing, or let himself get out of his own hands. One must think to think, as well as to work; and when he stops thinking what he will think, he stops thinking altogether. Thoughts no more go on without consideration than deeds.

We must think often to get a good thought; and oftener to get enough good thoughts to count; and we must think on many subjects to think well on one. Too many are thinking for thoughts to have much value unless they are many and strong.

We must look wide around to find anything good, and look wherever openings exist for thought. Life's opportunities lie, like gold, in small crevices which elude careless eyes. The best is generally where none look for it.

Men must look often to see; and they should keep thinking till they view enough things to find the best, which a few may not contain. Great thinkers have most to think from, and so more chances to get the best; and we should get the best of many things instead of the best of a few. Most that strikes the mind, like most that strikes the eye, is not worth noticing, and we should think often enough to get the exceptional good.

If we have not thought right we should think again, and differently. Few learn to think all the ways they can. The man of many kinds of thought is the successful man. Thoughts too much alike count for but one. and we should not only keep thinking, but change thinking. To think always the same is to stop thinking, variety of thought alone being thought. Thinkers are all original thinkers, whereas repeated thought is thoughtlessness. A world of great variety cannot be disclosed to thoughts of much similarity.

He never does better who always does as he has done; and if he finds nothing new he should find new ways of thinking. As all want something else, they should do something else. Men may not do what they have done if they want what they have not.

We are measured by the height to which our thought rises. To think is to be most of a man; and we should live chieffy in his head. Since everything issues from the brain, it should be made right there.

We must think that we may think. One thought follows another, and some thoughts are not possible till after others; and if the first do not appear, the latter do not come. Thought runs through time, and must be long, as well as constant, to reach full proportions. We need trains, as well as single thoughts, and should complete the series. Each thought suggests more, and should be followed as far as it will go, and exhaust our capacity as thinkers.

Men think little till they think in the consequences of their thought; and in going farthest in thought-implications they get most of the world. For we take up into ourselves as much as we know, which alone we use. The thinker is master from the mere fact of having a thought which is complete only on achieving a success in some result. For thought turns to action before reaching its consummation, and attends that action to the realization of the ideal.

Few think all the time, or all they can. There are lazy thinkers as well as lazy actors. Some think one thought a day, and spend the rest of their time working on it. Some think only in youth, and keep the opinions then formed for subsequent rumination. The successful are they who keep up their thought through life, and often think to their greatest depth.

Only by thinking are we resolved into power. Our energies then go to the head and heart, and we are set into action; whereas without thought our forces slumber, and produce but waste.

We should be alive as long as we can, and as much alive as we can. He lives most who thinks most, and lives longest who thinks to most purpose. We should not too early make acquaintance with death; and stagnation is mild death.

We should learn to think through our whole mind. By getting all our thoughts at work we are doubly thinkers. The greatest power of thought is far from the lowest, the measure of a man being a long one. Ability to use the whole mind is rare, whereas all the mind, like all the lungs, should often be called into play. Consumption of intellect comes from inaction of parts of it. One knows not how large he is till his mind rises to its full height.

We should not only think at our best, but think often at our best. Success depends on the amount of mind put on projects, and we should learn to be habitually great. The power to do our best is the condition of success, and especially to keep long at it. We never do our best at once. He who can work through time is the great accomplisher To be a great man longest is to be the greatest man. Magnitude runs through time as well as space.

We should take time to think. Many fail to do this because they are always working. We cannot be too busy to think, but should be most busy in thinking. We always have time for the most important, which is head-work.

Instead of beginning a second task when one is done, we should think between efforts. We cannot do our intellectual best at one thing any more than at one time, but must take in its relations for great thoughts.

We should think hard enough, often enough, and long enough to devise all of which we are capable. Neither capacity nor time should be wasted. If we did more thinking we might do less working. For thought often does our work for us, or dispenses with it.

We should occasionally go apart to ourselves and call up all of ourselves. Only when alone have we the company of the whole world, and we should learn to turn into moments of all thought, and be often at our greatest.

We should compel ourselves to think. Few incline to thought spontaneously. We must force ourselves into our highest altitude, though climbing is irksome. Getting control of what starts thoughts, we should keep them going. The intellect must be backed by the will. When mind stops, man stops; and only in needed rest and sleep should we consent to be nothing.

Especially should we compel ourselves to think great things, and to persist therein. This is necessary if we do them. We should learn to start up, to start hard. and to maintain speed. The time we can keep at our greatest is the measure of our ability, and our power to force this condition on ourselves determines our efficiency. The best of a man should do what he has to do, and he should make all of himself available. To turn a whole man into something is to make a great result.—Austin Bierbower, in *Success*.

